# Ignorance and Ill Health

**Published:** 1877-09

**Authors:** 


### IGNORANCE AND ILL HEALTH.

A lady correspondent, for weary months and years an invalid, writes in reference to a sentiment advanced in one of the Journals—that sickness is the result very generally of ignorance or inattention—says :

"I am willing to let it be, that my illness is the result of ignorance, but, I suppose, if the whole were known, it would be seen that some of my ailments are inherited. I was early taught that if I disobeyed the physical law, illness would be the result. My father was a physician, a regular graduate, but died while I was young, and in a few years I had a new one to care for me, consequently I did not have that kind of training which so nervous a child as I ought to have had ; under more favorable circumstances I might have had better health.

"If I had my life to live over again. I should select such occupations as would be most conducive to health, even if it were all drudgery. Inclined to be ambitious, and not having the means to enable me to " take the world easy," I hurried on, by night and day, until I could do so no longer. We often think in youth, if we could accomplish a desired object, our happiness would be complete, but when attained we are not happy, and sigh for something else. The only thing I sigh for now is good health, and if ever I come into possession of so great a happiness, it will be highly prized."

Such, reader, is the experience of multitudes, learned at a time of life too late for remedy ; have a care then that such may not be yours; let the promotion of your health be a prominent ingredient in every plan and every avocation, and you will feel thankful for the result to the latest hour of your life, for it bears a value far beyond that of glory or of gold.

